# A pharmacoeconomic approach to assessing the costs and benefits of air quality interventions that improve health: a case study

**DOI:** 10.1136/bmjopen-2015-010686

**Published:** 2016-06-21

**Authors:** James Lomas, Laetitia Schmitt, Sally Jones, Maureen McGeorge, Elizabeth Bates, Mike Holland, Duncan Cooper, Richard Crowther, Mike Ashmore, David Rojas-Rueda, Helen Weatherly, Gerry Richardson, Laura Bojke

**Affiliations:** 1 University of York, Centre for Health Economics, York, UK; 2 Academic Unit of Health Economics, University of Leeds, Leeds, UK; 3 City of Bradford Metropolitan District Council, Bradford, UK; 4 Improvement Academy, Yorkshire and Humber Academic Health Science Network, UK; 5 City of York Council, York, UK; 6 Ecometrics Research and Consulting, Reading, UK; 7 Wakefield Council, Wakefield, UK; 8 Leeds City Council, Leeds, UK; 9 CREAL (Centre de Recerca en Epidmiologia Ambiental)

## Abstract

**Objective:**

This paper explores the use of pharmacoeconomic methods of valuation to health impacts resulting from exposure to poor air quality. In using such methods, interventions that reduce exposure to poor air quality can be directly compared, in terms of value for money (or cost-effectiveness), with competing demands for finite resources, including other public health interventions.

**Design:**

Using results estimated as part of a health impact assessment regarding a West Yorkshire Low Emission Zone strategy, this paper quantifies cost-saving and health-improving implications of transport policy through its impact on air quality.

**Data source:**

Estimates of health-related quality of life and the National Health Service (NHS)/Personal Social Services (PSS) costs for identified health events were based on data from Leeds and Bradford using peer-reviewed publications or Office for National Statistics releases.

**Population:**

Inhabitants of the area within the outer ring roads of Leeds and Bradford.

**Main outcomes measures:**

NHS and PSS costs and quality-adjusted life years (QALYs).

**Results:**

Averting an all-cause mortality death generates 8.4 QALYs. Each coronary event avoided saves £28 000 in NHS/PSS costs and generates 1.1 QALYs. For every fewer case of childhood asthma, there will be NHS/PSS cost saving of £3000 and a health benefit of 0.9 QALYs. A single term, low birthweight birth avoided saves £2000 in NHS/PSS costs. Preventing a preterm birth saves £24 000 in NHS/PSS costs and generates 1.3 QALYs. A scenario modelled in the West Yorkshire Low Emission Zone Feasibility Study, where pre-EURO 4 buses and HGVs are upgraded to EURO 6 by 2016 generates an annual benefit of £2.08 million and a one-off benefit of £3.3 million compared with a net present value cost of implementation of £6.3 million.

**Conclusions:**

Interventions to improve air quality and health should be evaluated and where improvement of population health is the primary objective, cost-effectiveness analysis using a NHS/PSS costs and QALYs framework is an appropriate methodology.

**Strengths and limitations of this study**

- Applying cost and quality-adjusted life year (QALY) methods from economic evaluation in healthcare allows the assessment of cost-effectiveness from a healthcare perspective and facilitates comparison with other health interventions.
- The methods outlined above will allow local authorities to prioritise low emission strategies (supported by health and economic benefits) alongside other public health interventions.
- A fuller consideration of uncertainty would take in to account the following stochastic elements: estimated reduction in emissions, modelled dispersion, health effects of exposure to air pollutants, values of health impacts and assumptions made in generating estimates of QALY.
- The analysis also only assesses one intervention (the introduction of pre-EURO 4 buses and HGVs are upgraded to EURO 6 by 2016). However, decision-makers are likely to be faced with a multitude of competing alternatives, both air quality interventions and other public health interventions.

## Introduction

In total, 40 000 deaths are attributable to exposure to outdoor air pollution, each year in the UK.^1^ As a result, there is an increasing interest in studying the relationship between air quality and health, and the availability of evidence to support this association is accumulating;^2–4^ with air pollution having been described as ‘the public health problem that won't go away’.^5^ Interventions aimed at improving air quality incur costs and generate benefits. Establishing the value for money of these interventions is a crucial criterion for policymakers' decision-making. In particular, it is important that value for money is established in a manner that is consistent with other types of intervention to facilitate comparison, for example, between a strategy that improves air quality and the introduction of a smoking cessation campaign. This can ensure that the most cost-effective policies are implemented to maximise population health given fixed, or even shrinking, budgets.

To determine cost-effectiveness, we can look to the methods used by the National Institute for Heath and Care Excellence (NICE), which is responsible for making recommendations for health-improving interventions in the UK. NICE has substantial experience in employing cost-effectiveness evidence to determine appropriate guidance on the use of pharmacological treatments, for example, in its decision to not recommend β-interferon and glatiramer acetate for the treatment of multiple sclerosis in 2002.^6^ Recently, NICE's remit has extended to include guidance on public health interventions for which evidence is also centred on the estimated cost-effectiveness, for example, guidance on interventions to promote smoking cessation.^7^ While NICE develops guidance specifically for England and Wales, its evidence is often seen internationally and there are similar organisations that exist in other countries, for example, PBAC (Australia), CADTH (Canada) and HITAP (Thailand) to name but three. The process employed by NICE is rigorous and accountable, and by employing a generic measure of health, quality-adjusted life years (QALYs, note that another generic measure of health, disability-adjusted life years (DALYs), has been proposed for use with air quality,^8^ ^9^ but are not discussed here given that it is not used by NICE), it ensures that estimates of cost-effectiveness from different types of health-promoting interventions are comparable and decision-making consistent. For this reason, this paper explores the use of a QALY approach to health valuation and cost-effectiveness analysis to compare interventions that reduce exposure to poor air quality.

This research is timely for two reasons. First, NICE is working towards a set of guidance regarding outdoor air quality and health anticipated to be published in June 2017. And second, air pollution (and in particular road-traffic pollution) is a major concern in cities throughout Europe and in particular, the UK. The European Commission and the Supreme Court has issued a challenge to the Department for Environment, Food and Rural Affairs (DEFRA) to ensure full compliance with nitrogen dioxide (NO_2_) limit values by 2025. In order to achieve this, plans for the introduction of ‘clean air zones’ (CAZs) have been developed throughout the UK. The CAZ strategies implemented will have similar types of health outcome to ‘low emission zones’ (LEZs) such as the West Yorkshire LEZ (WYLEZ) analysed in this paper. While the proximal motivation for CAZs may be to achieve compliance, there may well be significant cobenefits in terms of generating health and, indeed, it may be the case that these interventions are cost-effective, in terms of health, in their own right.

NICE public health guidance^10^ describes the potential methods for the conduct of an economic analysis of public health programmes including cost-effectiveness analysis, cost-utility analysis, cost–benefit analysis and cost-consequence analysis. While this guidance demonstrates the pros and cons of each type of analysis, it is not prescriptive in which method is preferred. Where most of the benefits of an intervention are in the health sector, it is appropriate to utilise techniques from the economic evaluation of heath technologies,^10^ a form of cost-effectiveness analysis. We consider the use of QALYs in economic evaluation as a means to quantify any health impact and provide a basis for calculating the value for money of air quality interventions, both in theory and by example through an application of our estimates to the case study of a policy scenario of the WYLEZ compared against a ‘do nothing’ scenario.

## Methods

### Overview

Following the approach taken by NICE for evaluating health technologies,^10^ only National Health Service (NHS) and Personal Social Services (PSS) costs are considered in this analysis, with costs falling outside of these budgets not taken into account, although we compare our final estimate of health-related benefits to the implementation cost of the scenario under consideration that does not fall on the health sector budget. Health benefits are quantified by using the QALY: a composite measure of number of life years and the quality weighting associated with this (health-related quality of life (HRQoL)). The QALY is anchored at 0 (assumed to represent death) and 1 (assumed to represent full health). NHS/PSS costs and QALYs are modelled over the lifetime of the affected individuals' lifetime and those falling in future years are both discounted at 3.5% per annum.^11^ Alternative interventions can then be compared in terms of their cost and QALY profiles and incremental cost-effectiveness ratios (ICERs) can then be used to assess cost-effectiveness by comparing this ICER with the threshold value of a QALY.^10^ The threshold value of a QALY represents the exchange rate between the costs and the effects that is the rate at which a healthcare decision-maker is willing to substitute health outcomes and resources. It represents the opportunity cost of the health foregone in other areas, by adopting a new technology. While a full discussion of the underlying theory behind the cost-effectiveness threshold is beyond the scope of this paper, it is important to clarify that it is based on an estimate of the health opportunity cost of healthcare expenditure (because of constraints such as that imposed by a fixed budget), rather than the consumption value of health (for which estimates of value can be obtained through methods such as willingness to pay (WTP) or value of a statistical life (VSL)). Alternatively the threshold value of a QALY can be used in order to provide valuations in terms of net monetary (or net health) benefit.^12^

There is a burgeoning evidence base surrounding causal links between various health effects, in particular those affecting chronic morbidity,^13–17^ and poor air quality. Replicating WTP surveys to maintain up-to-date valuations of air quality impacts is expensive and time consuming (an example survey question is provided in the online supplementary appendix). Instead, quantifying the benefit associated with air pollution improvement interventions in terms of QALYs and applying an associated threshold value for a QALY gained provides a flexible and generalisable approach to estimating the impact of air quality on health and healthcare costs. QALYs are less frequently used in environmental economics in favour of WTP and VSL methods, but have great precedent in health economics for quantifying health effects.^18–23^ QALYs can also be used as part of cost–benefit analysis, in which case it may be desirable to value QALYs using their social consumption value.^24^

10.1136/bmjopen-2015-010686.supp1 Supplementary appendix

### The WYLEZ case study

The West Yorkshire Zone, as classified by DEFRA, has the fourth most significant NO_2_ concentration issues in the UK (after London, West Midlands and Greater Manchester). In addition, 5.4% of all mortality in West Yorkshire in 2010 was attributable to exposure to atmospheric particles smaller than 2.5 μm in aerodynamic diameter (PM_2.5_)_._^25^ In 2011, Bradford Metropolitan District Council (BMDC) and Leeds City Council (LCC) were awarded grants by DEFRA to undertake a LEZ feasibility study. The work has focused on establishing the benefits of low emission strategies that could result from cleaner bus, freight, taxi and private care fleets.

As part of the LEZ feasibility study, Cooper and colleagues conducted a health impact assessment (HIA). It is a public document and is available online at the following URL: http://www.bradford.gov.uk/NR/rdonlyres/1B122A0C-D989-451D-B0BE-30A46F0FF569/0/ReportOfTheLEZFeasibilityStudy.pdf. This HIA comprises several parts: modelling of traffic and emissions under different policy scenarios, dispersion modelling, a model to convert concentrations into lower layer super output area (LSOA)-level exposure and the application of response functions that generate estimates of changes in numbers of health events given changes in exposure and baseline levels of health events. Full details can be obtained from the document itself, but some key features for the purposes of this paper are described below.

The HIA looked only at the long-term health effects from exposure to PM_2.5_ and NO_2_. The health effects of other pollutants such as ozone were therefore not considered. In addition, the acute health effects of short-term exposure to air pollutants were not considered. These health effects are likely to be of a smaller magnitude than those considered^26^ and so would be unlikely to influence results. Estimates of the quantitative impact on health from exposure to poor air quality are taken from studies that were considered high quality either through being a meta-analysis identified through PubMed^13–17^ or by virtue of being a study that is heavily cited and recommended by COMEAP.^15^ The resulting studies, details regarding study type and their estimated effects on health are shown below in table 1.

**Table 1:** Exposure response functions from Cooper *et al*^30^

Health event	Pollutant	Reference	Study type	Type of effect	Exposure response function
All-cause mortality death	PM_2.5_	Pope, *et al*^15^	Cohort study (USA)	Annual	Relative risk of 1.06 (95% CI 1.02 to 1.11) per 10 μg/m^3^ increase in PM_2.5_ exposure
Coronary events (Bradford only)	PM_2.5_	Cesaroni, *et al*^13^	Meta-analysis of cohort studies (Europe, not including UK)	Annual	HR of 1.19 (95% CI 1.00 to 1.42) per 5 μg/m^3^ increase in PM_2.5_ exposure
Cases of childhood asthma	NO_2_	Takenoue, *et al*^17^	Meta-analysis (worldwide, not including UK)	Prevalence	OR of 1.135 (95% CI 1.03 to 1.25) per 18.8 μg/m^3^ increase in NO_2_
Term, low birthweight birth	PM_2.5_ and NO_2_	Pedersen *et al*^14^	Pooled cohort studies (Europe, including UK—born in Bradford)	Annual	OR of 1.18 (95% CI 1.06 to 1.33) per 5 μg/m^3^ increase in PM_2.5_ exposure and 1.09 (95% CI 1.00 to 1.19) per 10 μg/m^3^ increase in NO_2_
Preterm birth	PM_2.5_	Sapkota, *et al*^16^	Meta-analysis (worldwide, not including UK)	Annual	OR of 1.15 (95% CI 1.14 to 1.16) per 10 μg/m^3^ increase in PM_2.5_ exposure

PM_2.5_, atmospheric particles smaller than 2.5 μm in aerodynamic diameter.

In certain cases, more recent meta-analyses have become available and more widely used than those used in the original HIA, for instance all-cause mortality due to PM_2.5_ exposure in ref. 27. In addition, more health conditions have been linked with poor air quality exposure, without a meta-analysis having been performed (eg, see ref. 28, for incidence of bronchitis). In this paper, the links used in the published HIA are taken as given. The purpose of the paper is to apply the NICE framework and to demonstrate the application of a new methodology to the field of air quality using a real-world case study. The paper explores the use of cost-effectiveness analysis to inform local decision-making in WYLEZ, working with locally available data where possible. As can be seen in table 1, only one of the health events is modelled as a response to exposure of both pollutants. Therefore, any double counting through modelling term, low birthweight births as a response to both pollutants independently is likely to have only a small impact on results obtained. However, this is a bigger consideration in the broader literature where many of the same outcomes can result from PM_2.5_ and NO_2_ and very often people are simultaneously exposed to both (see for instance discussion in ref. 29). In addition, there may be synergistic effects arising from exposure to both that are not considered in the analysis here. The study also assumed that there was no time lag between exposure to pollutants and the resulting health events; this is again another simplification that should be taken into account when looking at the results (see ref. 26 for further details on issues around modelling this lagged effect of exposure).

In this analysis, the health effects of one intervention (pre-EURO 4 buses and HGVs were upgraded to EURO 6 by 2016) were taken from an analysis undertaken as part of the LEZ feasibility studies.^30^ ^i^ The comparison is with a 2012 baseline (no intervention).

The health effects of this scenario relative to no intervention were taken from LSOAs with centroids within the outer ring roads (ORRs) in Leeds and Bradford resulting from estimated reductions in emissions from within Leeds and Bradford ORRs of 13.502 t of PM_2.5_ per annum and 352.28 t of NO_x_ per annum (where 50% of NO_x_ is assumed to be NO_2_ for the purposes of the HIA). This is the change in emissions relative to the 2012 baseline that acts as the comparator in this evaluation, which results from the change in traffic-related emissions (background concentrations of pollutants are common to both scenarios and therefore do not affect the changes in exposure). Note that here a conservative assumption has been applied in terms of limiting the health effects to within the same area as impacted by the introduction of the LEZ. This underestimates the health impacts for two reasons. One is that a LEZ will affect traffic and so emissions and exposure in the surrounding area. The other is that emissions within the LEZ are dispersed and so may cause exposure outside of the LEZ itself. Both of those would be captured in an ideal analysis, but in this paper, we apply a practical simplification that represents a lower bound on the health effects of the policy.

### Estimating the NHS/PSS costs and QALYs associated with air quality-related health endpoints

Obtaining estimates of HRQoL and NHS/PSS costs for each of the events in the HIA requires some assumptions, which are detailed elsewhere.^31^ An example of such an assumption pertains to the conversion of attributable deaths to QALYs. According to ref. 26, exposure to PM_2.5_ leads to roughly 29 000 attributable deaths in the UK and 340 000 life years lost. We therefore assume that 11.72 life years are lost for each attributable death. In addition, we assume that each person affected loses 2 years of life (5.86 people affected per attributable death) and that they were in 75+ age category (HRQoL for this age group is 0.73 on average according to ref. 32). After discounting, the health loss associated with an attributable death is 8.4 QALYs. A summary of the sources used is provided in table 2.^ii^

**Table 2:** Data sources for cost and quality-adjusted life year estimates

Health event	Sources used for health cost calculation
All-cause mortality death	32 26
Coronary events (Bradford only)	32–37
Cases of childhood asthma	32–35 37–41
Term, low birthweight birth	33
Preterm birth	33 32 42 43

For each of the health impacts estimated from the HIA, the QALYs lost and additional NHS and PSS resources used were evaluated. The central estimates of the value of these health impacts when following this approach (and valuing a QALY at £20 000) are given in table 3. Full details of their calculation can be found in ref. 31 (see online supplementary appendix for full URL of this publication). The inclusion of QALYs and NHS/PSS costs is consistent with a pharmacoeconomic approach to cost-effectiveness analysis, such as that typically adopted by NICE for the evaluation of interventions where costs largely fall on the health sector.

**Table 3:** Summary of NHS/PSS costs and QALYs associated with each case of the health end points from the HIA

Health outcome	QALY loss, one decimal place	Additional costs, nearest £1000 (£ 2013/2014)	Combined loss, nearest £1000 (£ 2013/2014)
All-cause death	8.4	–	£168 000
Coronary event	1.1	£28 000	£50 000
Term, low birthweight birth	−	£2000	£2000
Preterm birth	1.3	£24 000	£50 000
Childhood asthma	0.9	£3000	£21 000

HIA, health impact assessment; NHS, National Health Service; PSS, Personal Social Services; QALY, quality-adjusted life year.

## Results

In this section, we present the results for the valuation of this LEZ scenario using the approach discussed in the previous section. The results from these are summarised in table 4.

**Table 4:** Summary of results

Health event	Pollutant	Number averted per year by implementing pre EURO 4 buses and HGVs were upgraded to EURO 6 by 2016	Total value per year, nearest £10 000 (£ 2013/2014)
All-cause mortality death	PM_2.5_	10	£1 680 000
Coronary events (Bradford only)	PM_2.5_	5	£250 000
Term, low birthweight birth	PM_2.5_	7	£20 000
Term, low birthweight birth	NO_2_	10	£20 000
Preterm birth	PM_2.5_	2.2	£110 000
Total annual effect			£2 080 000
Cases of childhood asthma	NO_2_	157	£3 300 000*

*Not annual effect but rather a one-off reduction in cases due to reduced prevalence.

NO_2_, nitrogen dioxide; PM_2.5_, atmospheric particles smaller than 2.5 μm in aerodynamic diameter.

The HIA considers more health outcomes than mortality, with morbidity cases averted forming a substantial proportion of the value of health benefits generated from reducing exposure to traffic-related air pollution. These include neonatal complications arising from air pollution as well as NHS/PSS costs of childhood asthma cases. The single largest component of the annual effect is from the impact of reduced PM_2.5_ on reduced all-cause mortality where 10 equivalent deaths are averted. These are valued at £1 680 000 per year. This constitutes over three-quarters of the total annual effect (the weight attached to mortality vs morbidity is variable among competing methodologies, as can be seen in ref. 44, where the percentage due to mortality is higher when VSL applied to attributable deaths compared with value of life year applied to life years). The remainder of the annual effect is made up of morbidity effects of other health impacts averted through reduction in exposure to PM_2.5_ (and some mortality effects due to pre term birth). Within the annual effect, roughly 1% is made up of health impacts averted due to reduced exposure to NO_2_. However, there is a substantial one-off value of £3 300 000 attached to the reduction in prevalence of childhood asthma that results from reduced exposure to NO_2_.

## Discussion

This paper highlights the potential to apply a method for assessing the likely cost-effectiveness of interventions to promote air quality that has been developed for the assessment of pharmacoeconomic interventions, including those appraised by NICE. Our results indicate an estimate of the annual value of the health impact of this intervention of £2 080 000 per annum alongside a one-off effect on prevalence of childhood asthma worth £3 300 000. According to ref. 21, the net present value of the cost of implementing this scenario is £6 300 000. Thus, the intervention appears to be cost-effective if differences in exposure are maintained for a sufficiently long time period (roughly 1 year and 5 months assuming discount rate of 3.5%).^iii^ However, a more careful consideration of alternative interventions should be used to inform a policymaker and the focus of this paper is methodological and the application is illustrative.

The use of cost-effectiveness analysis using QALY as an outcome measure is common in health technology assessment. Though there are examples of a QALY framework being applied to evaluate air quality interventions,^45–47^ these are relatively rare. While the advantages of the NHS/PSS costs and QALYs approach are substantial, allowing the comparison across a variety of interventions in different conditions, the approach also has its limitations and difficulties in public health evaluations.^48^ At present, the NHS/PSS costs and QALYs approach is used primarily where the perceived benefits are in the health sector. Ongoing work^49^ is exploring the potential for extending the health sector costs and QALYs approach to a multisectoral environment. However, this work remains in its infancy and approaches that allow the comparison of outcomes across several sectors (such as the damage cost method) have been preferred where substantial effects extend beyond health.

Where interventions to improve air quality are primarily aimed at improving health, should they not be compared with other health interventions, so that scarce resources can be used most efficiently? In these instances, there appears to be little justification to exclude air quality interventions from comparison with other ‘health’ interventions. In order to facilitate this comparison, the use of QALYs is desirable, which has been illustrated successfully in this paper.

### Strengths

Applying cost and QALY methods from economic evaluation^iv^ in healthcare allows the assessment of cost-effectiveness from a healthcare perspective and facilitates comparison with other health interventions. There is currently a robust health economic evidence base supporting some key public health interventions (eg, smoking cessation, alcohol screening and brief interventions, prescribing exercise classes).^v^ Recent research by COMEAP and others has demonstrated how poor air quality should join the list of public health priorities. Looking at England only, there were 80 000 deaths due to smoking among adults over 35 in 2013^50^ and 6500 deaths related to alcohol in 2012.^51^ This compares with 40 000 attributable deaths per year in the UK.^1^ The methods outlined above will allow local authorities to prioritise low emission strategies (supported by health and economic benefits) alongside other public health interventions.

### Limitations

It is worth noting that it may not always be possible to estimate an effect of a health event on QALYs or NHS/PSS costs due to data limitations or potentially a lack of sensitivity in the instrument for measuring health. One such example was our inability to estimate a QALY loss associated with term, low birthweight birth. In such cases, it is worth considering the total health effects that can be assigned a QALY loss as potentially a lower bound, since others may exist but cannot be valued for one of the reasons above. It is also worth noting that if the issue is that the health effect is too small to be detected, then it is unlikely to have a large bearing on decision-making.

The results presented above only consider a point estimate for the effects of the LEZ scenario, this masks the large uncertainty associated with each of the estimated components. One such uncertainty arises from the length of lag between exposure and health effect. Indeed this is one aspect of uncertainty, but there are many more that are relevant to the decision-maker. Presenting only the point estimates is useful for a comparison of the expected values generated by the LEZ scenario. A fuller consideration of uncertainty would take in to account the following stochastic elements:

1. Estimated reduction in emissions;
2. Modelled dispersion;
3. Health effects of exposure to air pollutants;
4. Values of health impacts;
5. Assumptions made in generating estimates of QALY.

The analysis makes only one comparison between the introduction of pre-EURO 4 buses and HGVs are upgraded to EURO 6 by 2016 and a preintervention (2012) baseline. However, decision-makers are likely to be faced with a multitude of competing alternatives, both air quality interventions and other public health interventions. Thus, a more useful analysis would include an assessment of the cost-effectiveness of several relevant comparators rather than comparing one intervention with a preintervention baseline. Importantly, the principle of using a common measure of benefit (such as QALYs) is crucial for aiding comparison. There are projections of how the transport fleet may evolve that is often referred to as a ‘do nothing’ scenario, which is the preferred comparator in ref. 21. However, it is potentially preferable to use the preintervention baseline if the assumptions underlying the projection are questionable. For example, enforcing the ‘do nothing’ projections may not be without cost if neighbouring regions implemented a LEZ and the associated consequences in terms of relocation of older vehicles within the national transport fleet. The purpose of the case study is to illustrate the cost-effectiveness methodology using QALYs as a measure of health benefits, based on NICE guidance. We only claim that the illustration is generalisable to the extent that the health effects considered would be common to similar types of interventions under consideration. These would include CAZs across UK and therefore this paper will be useful to local decision-makers across the country. However, there are many specific features of the case study and the interested reader may wish to investigate other similar studies if they are interested in the evaluation of air pollution policy more generally (see for instance refs. 22, 23 and 52).

In the context of a transport intervention, further costs may be of interest, for instance those that fall on bus companies, or on council-provided services or sectors such as the educational sector. The illustration in this paper provides valuable information on the health sector consequences, but further research on the impacts on other sectors would be complementary and valuable.

## Conclusion

Interventions to improve air quality and health should be evaluated and where improvement of population health is the primary objective, cost-effectiveness analysis using the NICE reference case with NHS/PSS costs and QALYs framework is an appropriate methodology. Alternative methodologies exist such as cost–benefit analysis, which has greater precedence in the environment literature. It should be noted that NHS/PSS costs and QALYs can be used as an input into cost–benefit analysis also.

## Footnotes

i Available for download as ‘Bradford Low Emission Zone Feasibility Study’ at http://www.bradford.gov.uk/bmdc/the_environment/pollution_noise_and_nuisance/air_quality.

ii Data that can be made available can be obtained by contacting the corresponding author (JL) at james.lomas@york.ac.uk. These data take the form of a spreadsheet in which calculations were made. Baseline data to which response functions were applied are not able to be made publicly available due to small numbers of counts.

iii The time horizon over which a decision-maker would take into account costs and benefits is clearly an important issue in this scenario, but this is true more broadly in terms of air quality, public health or even medical evaluations. In principle, the appropriate time horizon would be the period over which costs and benefits are different for comparators under consideration. In practice, however, one might adopt a time horizon in keeping with the specific policy under consideration, for example, the expected longevity of new buses.

iv It should be noted that we use the term evaluation, whereas the more precise wording might be *ex ante* evaluation, since we are constructing a model to evaluate the effects of an intervention before it has been implemented. *Ex post* evaluation refers to an analysis of an intervention after it has taken place, typically by examining changes in outcomes following its implementation.

v See NICE website: https://www.nice.org.uk/guidance/published?type=ph.
