# Supplementary material for: A pharmacoeconomic approach to assessing the costs and benefits of air quality interventions that improve health: a case study
Source: BMJ Open. 2016 Jun 21;6(6):e010686. doi: 10.1136/bmjopen-2015-010686 (PMC4916570; doi:10.1136/bmjopen-2015-010686)
Supplement: Supplementary appendix [file bmjopen-2015-010686supp_appendix.pdf]

# Appendix

## *Example question from Chilton et al. (2004)*

Survey respondents are asked to value in terms of their WTP, for example: “X MONTHS MORE LIFE IN NORMAL HEALTH. By reducing the general level of air pollution that causes wear and tear and faster ageing, everyone could live longer. That would mean that you {and everyone else in your household} could expect to live about X months longer in your {their} normal state of health.”

## *Estimated effects of policy scenarios on concentrations*

See report available at:
http://www.improvementacademy.org/documents/Projects/air_quality/Costs%20and%20benefits%20associated%20with%20emissions.pdf
